# Can a mobile application improve glucose-related and patient-reported outcome measures (PROMs) in people with type 1 diabetes mellitus? A randomized controlled trial using the mySugr^®^ app

**DOI:** 10.1007/s42000-024-00609-z

**Published:** 2024-10-16

**Authors:** Gemma Cuixart, Rosa Corcoy, Cintia González

**Affiliations:** 1 Medicine Department of Universitat Autònoma de Barcelona (UAB), Edifici M, Av. De Can Domènech, Bellaterra, 08193 Spain; 2 Research Institute of Hospital de la Santa Creu i Sant Pau, Carrer de Sant Quintí 77-79, Barcelona, 08041 Spain; 3 Fundació Hospital de l’Esperit Sant, Avinguda Mossèn Josep Pons i Rabadà, s/n, Santa Coloma de Gramenet, 08923 Spain; 4 Endocrinology and Nutrition Department of Hospital de la Santa Creu i Sant Pau, Carrer de Sant Quintí 89, Barcelona, 08041 Spain; 5 CIBER-BBN, Avenida Monforte de Lemos, 3-5. Pabellón 11. Planta 0, Madrid, 28029 Spain; 6 Endocrinology and Nutrition Department, Consorci Hospital General Universitari, Av. de les Tres Creus, 2, Valencia, 46014 Spain

**Keywords:** eHealth, Empowerment, mHealth, Mobile application, Telemedicine, Type 1 diabetes mellitus

## Abstract

**Purpose:**

Mobile applications (apps) have proven to be highly effective tools to empower patients with type 1 diabetes mellitus (T1DM) and enable them to achieve better self-care, quality of life (QOL), and glycemic control. The aim of the study is to examine whether mySugr^®^, an app for diabetes management, together with teleconsultations, can have a positive impact on these factors and, thereby, replace current clinical care.

**Methods:**

This study concerns an exploratory randomized clinical trial of 12 months’ duration. People with T1DM using multiple daily injections were randomized to usual care (bolus calculator, five face-to-face visits) or intervention (mySugr^®^ app, three face-to-face visits, and two teleconsultations). The main outcome was increase in empowerment assessed with the Diabetes Empowerment Scale Short Form questionnaire (DES-SF-S). Secondary outcomes were change in additional glucose-related (blood glucose monitoring, mean blood glucose, standard deviation, coefficient of variation (CV), and high and low blood glucose index) and patient-reported outcome measures (PROMs) (self-management, QOL, and distress).

**Results:**

A total of 25 out of 28 participants completed the study (52% men, age 44.52 years, diabetes duration 21.28 years). At 12 months, no significant differences were identified in the change of DES-SF-S and additional PROMs between arms. Similarly, no differences were observed in glucose-related outcomes except for the change in CV at 9 (control − 1.87 ± 4.98 vs. intervention 5.89 ± 11.33, *p* = 0.008) and 12 months (control − 2.33 ± 3.54 vs. intervention 5.12 ± 11.32, *p* = 0.018). Adherence to and satisfaction with the app were high.

**Conclusion:**

Patients with diabetes using the mySugr^®^ app and teleconsultation achieved similar results to those following usual care in empowerment, other PROMs, and most glucose-related outcomes, thus supporting its use in combination with face-to-face visits. The RCT was registered with ClinicalTrials.gov (NCT03819335, first registration 28/01/2019).

**Supplementary Information:**

The online version contains supplementary material available at 10.1007/s42000-024-00609-z.

## Introduction

Diabetes mellitus is among the most common chronic diseases, with an increasing worldwide prevalence, and is associated with morbidity, mortality (3% increase in diabetes mortality rates by age between 2000 and 2019), and a worse quality of life (QOL) [1]. In particular, persistently poor glycemic control in people with diabetes (PwD) is a common, complex, and serious problem which can lead to significant damage to the cardiovascular, renal, neural, and visual systems [2]. In the pursuit of improvement of metabolic control, we should focus on challenges related to insulin dose adjustment, blood glucose monitoring (BGM), and fear of hypoglycemia. Additionally, communication and coordination with a multidisciplinary team with shared goals and recommendations is relevant for patient self-care [3].

The COVID-19 pandemic and the restrictions implemented forced health systems to adjust the delivery of diabetes care, while patients and healthcare providers had to rapidly adapt to telemedicine in order to maintain social distancing [4]. The use of remote consultations was accepted by patients and perceived as positive by most of them [5], and, moreover, some studies observed that its use improved glucose control in children and adolescents with diabetes [6]. As a result, since the COVID-19 pandemic, technological tools that support self-management and communication with medical team have become more relevant than ever.

In this context, mobile phone technology is a powerful tool enabling provision of individualized healthcare with enough potential to support both patient decision-making and communication with medical teams. Currently, there are more than 150,000 health-related mobile applications (apps), most of them focused on wellness, nutrition, or exercise, but also for treatment and management of chronic diseases, such as diabetes. Apps have proven their usefulness in achieving health behavior goals and for medical care decision-making [7, 8].

In 2011, a meta-analysis documented an improvement in diabetes clinical outcomes related to apps use; however, the evidence was stronger among people with type 2 than among those with type 1 diabetes mellitus (T1DM) [9, 10]. Recently, another meta-analysis confirmed the effectiveness of apps in improving glycemic control, demonstrating a 5.4 mmol/mol (0.49%) HbA_1c_ reduction in people with T1DM [11]. On the other hand, the results of the included studies are heterogeneous, with four reporting no changes [12–15] and three observing significant differences [16–18]), so that the clinical utility of apps remains unclear. Nevertheless, the potential of apps to improve other health-related outcomes, such as empowerment, should be seriously borne in mind. When applied to diabetes, empowering patients means providing them with adequate knowledge, along with tools and techniques as well as support to implement these so that they can actively participate in the management of their health and become capable of taking appropriate decisions, in consultation with the medical team [19]. Healthcare facilities that incorporate empowerment-based diabetes management experience greater reduction in HbA_1c_, increase in glucose self-monitoring, and medication adherence [20]. Furthermore, one study evaluating the efficacy of an empowerment-based self-management consultant intervention suggests that treatments that focus on empowerment lead to improvements in diabetes-related QOL [19].

Similarly, growing evidence suggests that using apps may improve diabetes QOL and distress. Even though some studies observed no changes [15–18], others have reported improvement or borderline improvement for mental-health related QOL [14]. Specifically, a subgroup of 60 patients included in the Diabetes Interactive Diary study [12] had benefits on generic and diabetes-related scales of QOL; this finding was confirmed in a subsequent study [13].

Thus, with most studies focusing on HbA_1c_, the importance of an app could also reside in improving empowerment and QOL. Better self-management may result in more insight into the disease process, leading to better compliance and improved QOL, which is inversely associated with progression of disease, non-adherence to medication, and mortality [21]. Despite of that, an Australian studied reported that, regrettably, only a minority of adults with T1DM (24%) used certified apps to support their self-management [22].

Unfortunately, a systematic review of free apps in the English language showed that there were only nine out of 65 reviewed apps that were sufficiently versatile and useful for successful self-management of diabetes [23].

Therefore, bearing in mind the above, this study was initiated to investigate the effects of the mySugr^®^ app on empowerment of people with T1DM. We moreover investigated the impact on other glucose-related and patient-reported outcome measures (PROMs).

## Methods

### Study design and ethical considerations

This study was an exploratory, 12-month, randomized, open-label, parallel-group, single-center randomized controlled trial (RCT) in a tertiary care hospital in Barcelona, Spain.

The study protocol (Clinical Trials identifier NCT03819335), consent forms, and patient information sheets were approved by the Institutional Review Board of Hospital de la Santa Creu i Sant Pau (IIBSP-SUG-2018-01). All participants provided written consent before the study procedures were started.

The inclusion criteria were as follows: adults (≥ 18 years), T1DM with duration > 1 year, basal-bolus insulin regimen with multiple daily injections (MDI), last (< 3 months) HbA_1c_ ≥ 53 mmol/mol (7%) and < 75 mmol/mol (9%), knowledgeable about carbohydrate counting and functional insulin treatment, and with regular use of smartphone or tablet (android/iOS).

The exclusion criteria were as follows: use of an app for diabetes management at study entry, utilization of real-time or intermittent continuous glucose monitoring (CGM), being pregnant or planning pregnancy, and having any disease or clinical condition that might interfere with the study protocol (e.g., active cancer, severe mental disorder, or planning for surgery).

Participants were recruited at routine follow-up appointments in the Endocrinology Department. If they were eligible, after reading an information sheet and signing the informed consent form, the participants were randomized to the intervention group (IG) or the control group (CG), ratio 1:1, by one of the investigators (GC) using a sequence generated with https://www.sealedenvelope.com/ by a second investigator (RC). Allocation concealment was ensured.

The CG regularly used their glucometer Accu-Chek^®^ Aviva Expert bolus calculator (which provides bolus advice according to glycemia and carb intake) and were scheduled for five face-to-face visits with an endocrinologist (at baseline, 3, 6, 9, and 12 months). The IG were helped to download and install the mySugr^®^ app, received a compatible meter (which automatically uploads blood glucose values to the app via Bluetooth), and were instructed in their use. They were scheduled for five visits, three face-to-face (at baseline, 6, and 12 months) and two teleconsultations (at 3 and 9 months) using the mySugr^®^ Care web platform.

**Fig. 1:**
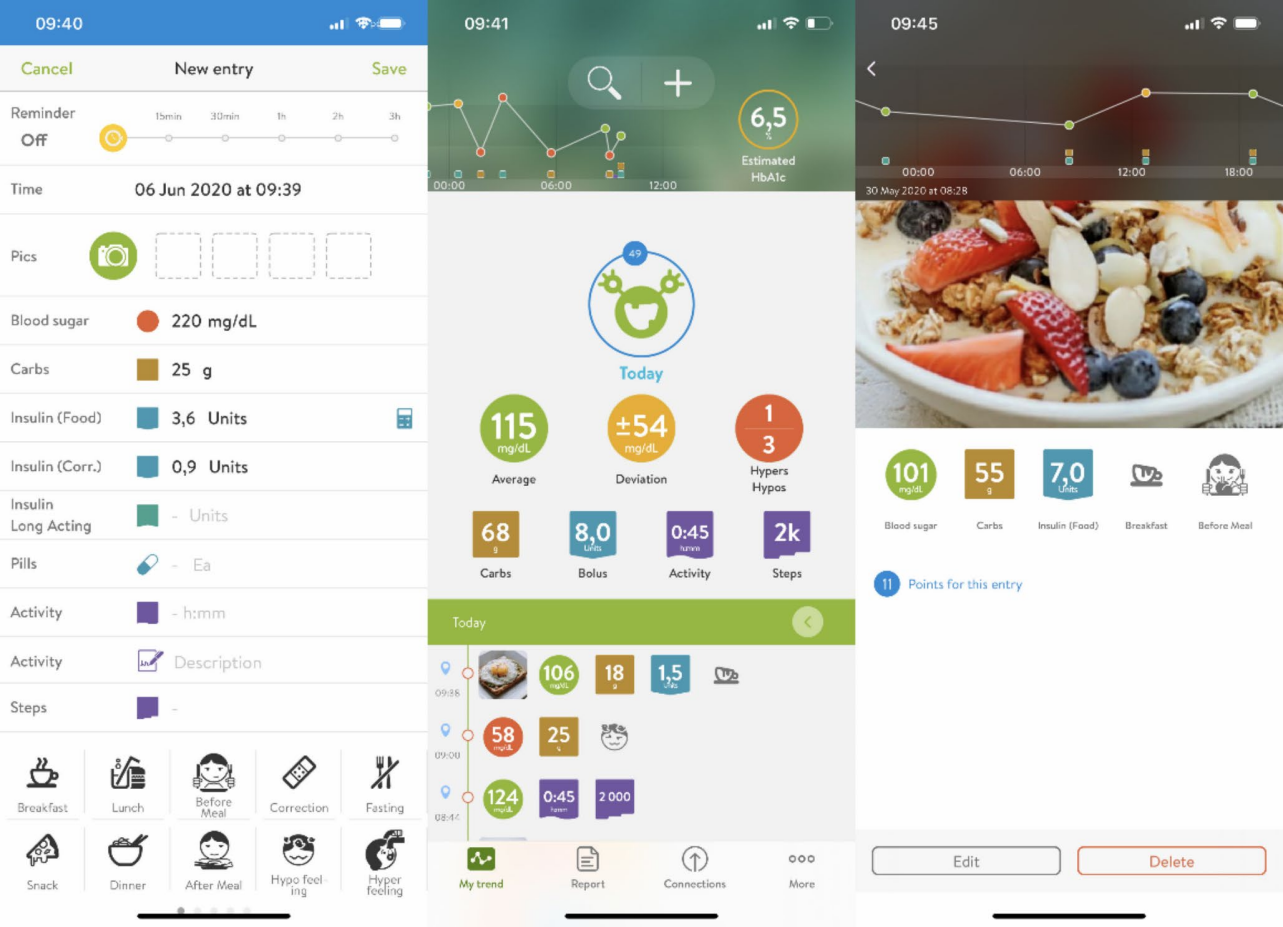
mySugr screenshots of diabetes diary and bolus calculator © 2021 mySugr GmbH

mySugr^®^ is an app for diabetes management from Roche Diabetes Care^®^ and has Free and Pro versions. Their features include a diabetes diary with automatic collection and analysis of data on glycemia (including estimation of HbA_1c_) and the possibility to manually enter information on food intake, physical activity, and insulin dose (Fig. 1). It is compatible with CGM systems and includes integration with Google Fit type motion sensors (to automatically collect physical activity data), a bolus calculator (activated for IG), reminders about BGM, and the possibility to save pictures of food consumed in the Pro version. The Pro version in the Spanish language was provided to the participants in the study.

We followed the Consolidated Standards of Reporting Trials (CONSORT) reporting guidelines (Supplementary Table 1).

### Data collection

At baseline and at the 12-month visit, patients were given the following questionnaires which they filled in: the Diabetes Empowerment Scale-Short Form Spanish version (DES-SF-S), diabetes-related tasks, the Diabetes Quality of Life Spanish version (EsDQOL), and the Diabetes Distress Scale Spanish version (DSS-S).

The DES-SF-S was used to measure psychosocial self-efficacy (Supplementary Table 2). The original DES has 28 items divided into three subscales, as follows: managing the psychosocial aspects of diabetes, assessing dissatisfaction and readiness to change, and setting and achieving goals [24]. The reduced version has free access and includes eight items, assessed through an ordinal scale of five answers [25]. The result is obtained after the sum of all items in which higher values are related to higher perceptions of psychosocial self-efficacy.

The diabetes-related tasks questionnaire was developed ad hoc to assess tasks related to and time requirement for diabetes self-management (Supplementary Table 3). It has five items rated in an ordinal scale from 1 to 4. A higher score means that the patient has the appropriate tools and needs less time and fewer tasks for diabetes self-management.

The EsDQOL questionnaire has 46 items which measure four domains that are highly relevant to treatment perceptions, as follows: satisfaction with treatment, impact of treatment, worry about social/vocational issues, and worry about the future effects of diabetes (Supplementary Table 4) [26]. All items are scored on a 5-point scale where a lower value means a better QOL. The Spanish version has been validated and has free access [27].

The DSS-S was used to assess diabetes-related distress (Supplementary Table 5). It is a free-access 17-item instrument with four subscales (emotional burden, physician distress, regimen distress, and interpersonal distress) where each item is rated on a 6-point scale. An average score of < 2.0 reflects little or no distress, between 2.0 and 2.9 moderate distress, and ≥ 3.0 high distress. A total or subscale score ≥ 2.0 is considered clinically significant [28].

Data regarding age, sex, highest level of education, employment status, body mass index, systolic and diastolic blood pressure, heart rate, diabetes duration, insulin therapy, and presence of diabetic complications were registered at baseline with information from electronic health records and via physical examination. HbA_1c_ was measured with a point-of-care method (DCA, Siemens DCA 2000+) at baseline and after 6 and 12 months.

At baseline and at all follow-up visits, the frequency of BGM, mean blood glucose (BG) and standard deviation (SD), coefficient of variation (CV), and high and low blood glucose index (HBGI and LBGI) were obtained from the glucometer download.

Moreover, patients in the IG filled in an ad hoc satisfaction questionnaire (Supplementary Table 6) and were asked if they still used the app after the end of the study.

Recruitment began in March 2019 and finished in February 2020, and the study ended in May 2021.

### Outcomes

The primary outcome was to compare the change from baseline to 12 months in empowerment, assessed after completion of the DES-SF-S questionnaire, between the IG and the CG.

Secondary endpoints were glucose-related outcomes, namely, change from baseline of mean BG and SD, CV, HBGI, and LBGI at each follow-up visit and change from baseline in HbA_1c_ at 6 and 12 months.

Other secondary endpoints included change from baseline in self-management (measured via the diabetes-related tasks questionnaire), change in QOL (evaluated with EsDQOL), and distress related to diabetes (evaluated with DSS-S), from baseline to endpoint, and change in adherence. Adherence was assessed with the rate of attendance at face-to-face visits and teleconsultations and, at each follow-up visit, the frequency of BGM and uptake of recommendations proposed (percentage of recommendations prescribed by the physician and accepted by the patient at the next visit). IG participants were also asked if they still used the app 3 months after the end of the study.

Finally, for IG participants, satisfaction with the mySugr^®^ app was assessed with a specific questionnaire (ad hoc) at the end of the study.

### Statistical analysis

The study was designed as an exploratory study so that a formal sample size calculation to test the hypothesis that mySugr^®^ app use with teleconsultation led to an improvement of empowerment at 12 months would not be performed. We did not use previous studies assessing empowerment [19] for calculation of sample size because they were performed in a different population and health system. Given that it was an exploratory study, the sample size was set at 15 patients per group. Considering that there could be a dropout rate of 10%, a minimum of 33 patients had to be enrolled.

Categorical data were expressed as frequencies and percentages. The Kolmogorov-Smirnov test was used to examine whether the quantitative data had normal distribution, and the data were expressed as mean and SD or median (P25, P75) accordingly. Differences were tested using the Mann-Whitney U test for non-normal distribution of continuous variables and Student’s T test for normally distributed continuous variables. Statistics were performed using IBM-SPSS version 26.0 (Chicago, IL). The data were analyzed according to the intention-to-treat principle.

## Results

### Patients

**Fig. 2:**
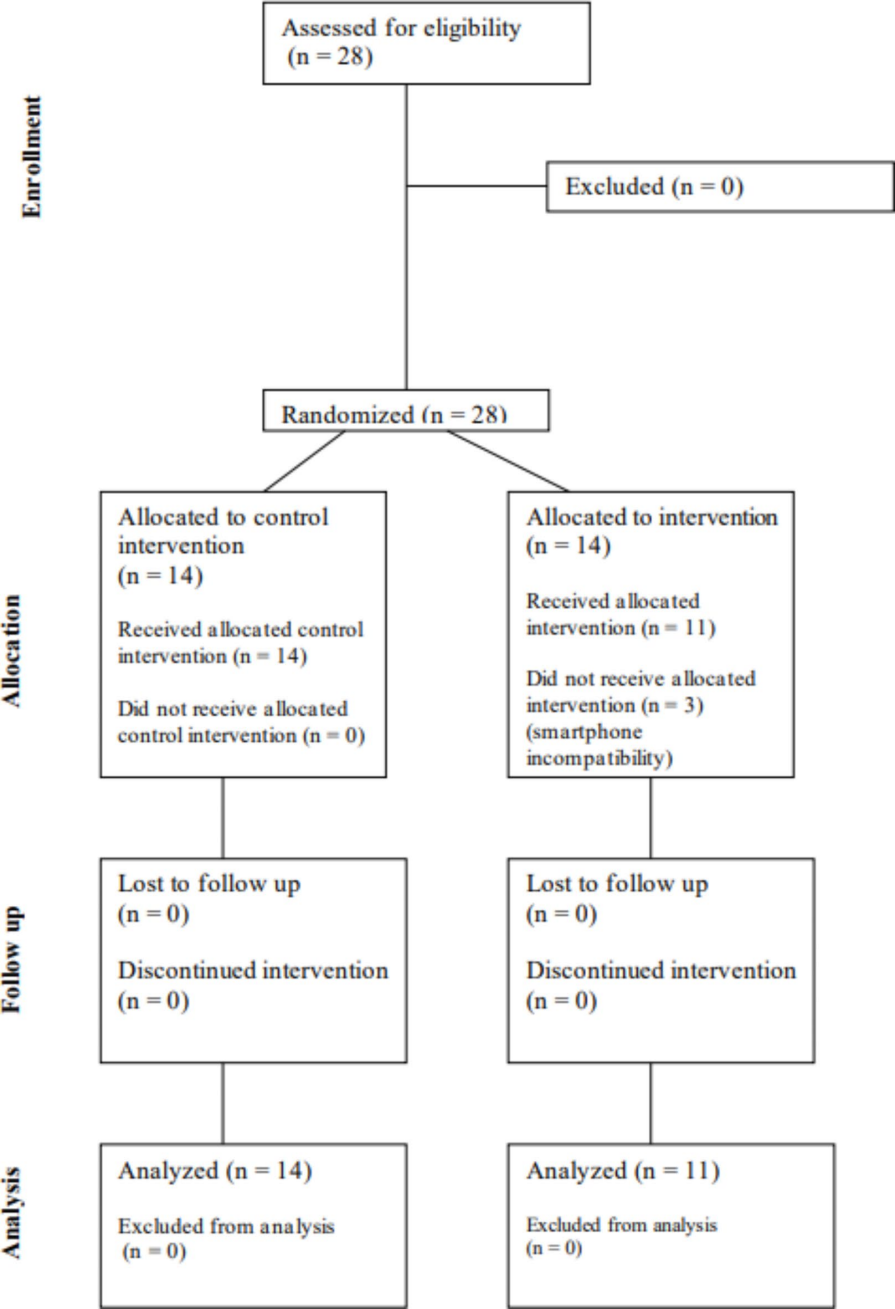
CONSORT diagram showing the flow of participants through each stage of the mySugr^®^ randomized trial

Recruitment was ceased before achieving the goal of 33 patients because of the COVID-19 pandemic. Of the 28 patients who were assessed for eligibility (14 in each group), three patients of the IG were excluded after randomization because of app-smartphone incompatibility (Fig. 2).

There were no significant differences between the two groups concerning any of the baseline characteristics (age, sex, highest level of education, employment status, body mass index (BMI), blood pressure, heart rate, duration and complications of DM, and HbA_1c_), except for the daily frequency of BGM, which was higher in the CG (4.56 vs. 3.50) (Tables 1 and 2). The characteristics of the included patients were as follows: age 44.5 years, 52% were men, BMI 27.4 kg/m^2^, diabetes duration 21 years, 32% presented associated complications, and average HbA_1c_ was 7.5%. There were no dropouts during follow-up.

**Table 1:** Participant characteristics at baseline

	All patients (*n* = 25)	Control group (*n* = 14)	Intervention group (*n* = 11)	*p*
**Age** (years)	44.5 ± 14.8	41.9 ± 15.0	47.8 ± 14.5	0.333
**Sex** (male)	13 (52)	7 (50)	6 (54.5)	0.821
**Highest level of school education completed**				0.632
Primary school	2 (8)	1 (7.1)	1 (9.1)	
Secondary school	17 (68)	9 (64.3)	8 (72.7)	
University	4 (16)	2 (14.3)	2 (18.2)	
Master/Doctorate	2 (8)	2 (14.3)	0 (0)	
**Employment status**				0.691
Student	1 (4)	0 (0)	1 (9.1)	
Employed	20 (80)	12 (85.7)	8 (72.7)	
Unemployed	2 (8)	1 (7.1)	1 (9.1)	
Retired	2 (8)	1 (7.1)	1 (9.1)	
**BMI**^**a**^ (kg/m^2^)	27.4 ± 4.6	26.9 ± 5.2	27.9 ± 3.8	0.605
**SBP**^**b**^ (mmHg)	122.8 ± 15.6	124.6 ± 15.1	120.6 ± 16.7	0.543
**DBP**^**c**^ (mmHg)	73.8 ± 9.1	74.6 ± 9.3	72.7 ± 9.2	0.627
**HR**^**d**^ (bpm)	74.7 ± 11.1	76.7 ± 11.3	72.2 ± 10.9	0.323
**DM** ^**e**^ **duration** (years)	21.3 ± 14.1	18.5 ± 11.2	24.8 ± 16.9	0.273
**Complications**	8 (32)	3 (21.4)	5 (45.5)	0.201
**HbA**_**1c**_**DCA** (mmol/mol, %)	58 ± 7.1 7.5 ± 0.65	58 ± 7.9 7.5 ± 0.72	58 ± 6.4 7.5 ± 0.59	0.975

*Abbreviations*: ^a^BMI, body mass index; ^b^SBP, systolic blood pressure; ^c^DBP, diastolic blood pressure; ^d^HR, heart rate; ^e^DM, diabetes mellitus

Data are presented as n (%) or mean ± standard deviation

**Table 2:** Glucose-related outcomes

		Baseline	Δ 3 months	Δ 6 months	Δ 9 months	Δ 12 months
**BGM**^**a**^ (nº/day)	Control (*n* = 14)	4.56 ± 0.32	0.15 (-0.33, 0.33)	-0.15 (-0.50, 0.15)	-0.15 (-0.43, 0.20)	-0.10 (-0.53, 0.13)
	Intervention (*n* = 11)	3.50 ± 0.38	0.00 (-0.40, 0.40)	-0.10 (-0.70, 1.40)	0.10 (-0.70, 0.90)	-0.10 (-0.90, 1.20)
	p	**0.043**	0.936	0.727	0.687	0.767
**Mean BG**^**b**^ (mmol/l)	Control (*n* = 14)	9.61 ± 0.42	-0.17 (-0.74, 0.21)	-0.50 (-0.75, 0.76)	-0.08 (-0.75, 0.35)	-0.25 (-0.61, 0.28)
	Intervention (*n* = 11)	9.82 ± 0.50	-1.00 (-1.61, 1.78)	-0.22 (-1.39, 1.11)	0.44 (-0.83, 1.22)	0.22 (-1.22, 0.72)
	p	0.757	0.467	0.609	0.317	0.467
**Mean SD**^**c**^ (mmol/l)	Control (*n* = 14)	4.14 ± 0.25	-0.14 (-0.46, 0.28)	-0.19 (-0.79, 0.35)	-0.22 (-0.69, 0.25)	-0.36 (-0.61, 0.03)
	Intervention (*n* = 11)	3.96 ± 0.29	-0.11 (-0.44, 1.11)	-0.06 (-0.44, 0.44)	0.17 (-0.33, 1.00)	-0.11 (-0.39, 0.89)
	p	0.635	0.767	0.403	0.085	0.085
**CV** ^**d**^	Control (*n* = 14)	43.3 ± 1.8	0.00 (-4.44, 2.18)	-1.41 (-5.97, 2.65)	-3.45 (-4.64, 1.18)	-2.34 (-5.78, 0.10)
	Intervention (*n* = 11)	39.9 ± 2.2	0.56 (0.17, 5.85)	-1.92 (-2.90, 8.62)	2.55 (-2.33, 11.87)	0.61 (-2.60, 8.93)
	p	0.241	0.134	0.403	**0.008**	**0.018**
**HBGI** ^**e**^	Control (*n* = 14)	10.3 ± 1.3	-0.80 (-2.00, 0.45)	-1.45 (-2.73, 1.55)	-0.20 (-2.80, 0.63)	-0.90 (-2.13, 0.73)
	Intervention (*n* = 11)	11.1 ± 1.6	-2.90 (-3.90, 5.20)	-0.60 (-5.20, 2.60)	0.50 (-3.60, 3.70)	0.60 (-4.60, 2.10)
	p	0.710	0.467	0.727	0.403	0.647
**LBGI** ^**f**^	Control (*n* = 14)	1.64 ± 0.29	0.25 (-0.60, 0.40)	0.20 (-0.45, 0.60)	-0.10 (-0.85, 0.35)	-0.10 (-0.30, 0.43)
	Intervention (*n* = 11)	1.25 ± 0.34	0.10 (-0.20, 0.30)	0.30 (-0.50, 0.50)	0.00 (-0.60, 0.60)	0.10 (-0.60, 1.10)
	p	0.383	0.893	0.936	0.434	0.727
**HbA**_**1c**_**DCA** (mmol/mol, %)	Control (*n* = 14)	58 ± 2.0 7.5 ± 0.18		1.01 (-3.28, 8.31) 0.1 (-0.30, 0.76)		-0.55 (-2.73, 5.79) -0.05 (-0.25, 0.53)
	Intervention (*n* = 11)	58 ± 2.2 7.5 ± 0.20		2.19 (-5.47, 6.6) 0.20 (-0.50, 0.60)		-2.19 (-7.65, 0.00) -0.20 (-0.70, 0.00)
	p	0.975		0.467		0.202

*Abbreviations*: ^a^BGM, blood glucose monitoring; BG^b^, blood glucose; SD^c^, standard deviation; CV^d^, coefficient of variation; HBGI^e^, high blood glucose index; LBGI^f^, low blood glucose index

Data are presented as mean ± standard deviation or median (P25, P75)

p-value ≤ 0.05 is considered statistically significant

### Empowerment, self-management, QOL, and distress

Change in empowerment from baseline to end point, measured with the DES-SF-S questionnaire, did not differ between groups (Table 3). Moreover, there were no statistical differences between the groups in other PROMs.

**Table 3:** Patient-reported outcome measures: diabetes empowerment, diabetes-related tasks, quality of life, and distress

Questionnaire (minimum-maximum score) ^*^Indicates worse questionnaire results		Baseline	Δ 12 months
**DES-SF-S**^**a**^ (8*-40)	Control (*n* = 14)	31.7 ± 1.0	1.00 (-0.25, 4.25)
	Intervention (*n* = 11)	30.7 ± 1.2	2.00 (0.00, 3.00)
	p	0.530	0.893
**Diabetes-related tasks** (5*-20)	Control (*n* = 14)	14.1 ± 0.5	1.00 (-0.50, 3.00)
	Intervention (*n* = 11)	13.9 ± 0.6	2.00 (-1.00, 5.00)
	p	0.840	0.467
**EsDQOL** ^**b**^			
Total (43–225*)	Control (*n* = 14)	86.7 ± 5.6	-1.50 (-9.50, 2.75)
	Intervention (*n* = 11)	90.1 ± 6.3	2.00 (-10.00, 11.00)
	p	0.692	0.501
Satisfaction (15–75*)	Control (*n* = 14)	32.5 ± 2.1	-1.00 (-5.25, 4.50)
	Intervention (*n* = 11)	32.8 ± 2.4	4.00 (-3.00, 7.00)
	p	0.922	0.434
Impact (17–85*)	Control (*n* = 14)	33.6 ± 2.4	-1.50 (-3.25, 2.25)
	Intervention (*n* = 11)	35.9 ± 2.7	0.00 (-6.00, 2.00)
	p	0.538	0.809
Social/vocational (7–45*)	Control (*n* = 14)	12.4 ± 1.4	-0.50 (-1.25, 0.25)
	Intervention (*n* = 11)	12.5 ± 1.5	-1.00 (-2.00, 2.00)
	p	0.990	0.893
Effects of diabetes (4–20*)	Control (*n* = 14)	8.14 ± 0.67	0.00 (-1.00, 1.00)
	Intervention (*n* = 11)	8.91 ± 0.76	0.00 (-1.00, 1.00)
	p	0.456	0.851
**DSS-S**^**c**^ (< 2.0 little or no distress, 2.0-2.9 moderate distress, ≥ 3.0 high distress)			
Total average	Control (*n* = 14)	2.08 ± 1.02	-3.00 (-9.75, 3.25)
	Intervention (*n* = 11)	1.91 ± 0.55	-2.00 (-6.00, 7.00)
	p	0.632	0.373
Emotional average	Control (*n* = 14)	2.30 ± 1.10	-1.00 (-4.00, 0.00)
	Intervention (*n* = 11)	2.33 ± 0.60	-2.00 (-3.00, 2.00)
	p	0.942	0.467
Physician average	Control (*n* = 14)	1.64 ± 1.22	0.00 (-1.50, 0.00)
	Intervention (*n* = 11)	1.32 ± 0.82	0.00 (0.00, 1.00)
	p	0.456	0.403
Regimen average	Control (*n* = 14)	2.27 ± 1.03	-1.00 (-4.00, 2.00)
	Intervention (*n* = 11)	2.20 ± 0.81	1.00 (-3.00, 2.00)
	p	0.853	0.434
Interpersonal average	Control (*n* = 14)	1.95 ± 1.46	0.00 (-1.00, 1.00)
	Intervention (*n* = 11)	1.52 ± 0.58	0.00 (0.00, 1.00)
	p	0.361	0.344

*Abbreviations*: ^a^DES-SF-S, Diabetes Empowerment Scale-Short Form Spanish version; ^b^EsDQOL, Diabetes Quality of Life Spanish version; ^c^DSS-S, Diabetes Distress Scale Spanish version

Data are presented as mean ± standard deviation or median (P25, P75)

p-value ≤ 0.05 is considered statistically significant

### Glucose-related outcomes

In terms of glycemic variability, we detected significant differences only in the change of CV at 9 and 12 months, favorable to the CG. Change in mean BG, SD, HBGI, and LBGI at 3, 6, 9, and 12 months and HbA_1c_ at 6 and 12 months did not differ between groups (Table 2).

### Adherence and satisfaction

A higher BGM frequency was observed in the CG at the beginning of the study and modifications during the study were similar in both groups (Table 2). Patients attended all face-to-face visits and teleconsultations and followed a similar percentage of physician recommendations (98.21% in CG and 97.72% in IG). Ten out of 11 IG participants still used the app 3 months after the end of the study. The IG scored the mySugr^®^ app in the satisfaction questionnaire with 17.55 ± 2.38 (0–20).

No important harms or unintended effects were identified in any participant.

## Discussion

In this randomized exploratory open-label controlled trial, people with T1DM using the mySugr^®^ app, together with teleconsultation, had a similar change in primary outcome (empowerment) and all secondary outcomes, except for CV.

This study is the second RCT evaluating diabetes-related empowerment using an app. A previous RCT in 72 T1DM people evaluated empowerment with the DES-SF questionnaire after 9 months using an app with text-message feedback from a Certified Diabetes Educator and no differences within and between groups were observed [17]. Our study differs in the absence of weekly feedback and addition to teleconsultation in the IG, this being designed with the intention of stimulating and supporting both the patients’ self-management and their problem-solving skills. Some previous web and mobile phone studies (using text messages) among PwD showed positive changes in empowerment [29–31], while others did not [32].

We did not detect differences in the change in QOL between groups over time. Several previous telemedicine studies in patients with T1DM were also unable to observe an improvement in QOL [15–18], while others evaluating apps reported either borderline [14] or distinct improvements in mental-health related QOL [12, 13]. The absence of changes in QOL could be because our patients already had good baseline QOL scores.

We did not observe differences in the change of the diabetes-related distress questionnaire during the study. A previous RCT in patients with T1DM was also not able to detect differences in emotional distress using a digital diabetes diary application [15]. As our study population had low baseline distress (total score, physician, and intervention subscales), the likelihood of improvement was limited.

The current study adds information on an important aspect of the mySugr^®^ app, namely, the fact that its use did not modify time and tasks related to diabetes self-management, any such modification potentially being important. This might be because mySugr^®^ automatically uploads glucose data from the glucometer, presents statistics of average glucose and estimated HbA_1c_, and facilitates users in searching for their historical data regarding past events [33].

Our study began in April 2019 and finished in May 2021. COVID-19 was first reported in Spain in January 2020 and, during this period, we experienced three waves, the first from March to June, the second from July to December, and the third from December to February 2021 [34]. There is growing evidence that the COVID-19 pandemic affected mental health and well-being, with an increase in mental health diseases and higher levels of anxiety and stress [35]. The pandemic may thus have influenced our study, affecting empowerment, QOL, and diabetes-related distress exerting, we assume, a similar effect in both groups.

In summary, we did not observe significant differences either in change in empowerment between groups, our main outcome, on other PROMs assessed or on most glucose-related outcomes.

Previous RCTs addressing app use in patients with T1DM showed differences in HbA_1c_ between the CG and the IG that ranged from − 1.17 to 0.10 [12–18], while a recent meta-analysis demonstrated statistically significant benefits, with an average reduction of 5.4 mmol/mol (0.49%) in HbA_1c_ that favored the intervention but exhibited considerable heterogeneity [11]. Preceding observational studies on usage of the mySugr^®^ app showed an improvement of HbA_1c_ from 75 (9%) to 61 (7.7%) after 6 months of use [36] and a reduction in mean BG, SD, HBGI, and LBGI [33]. The RCT herein reported did not observe an improvement in the six glucose-related outcomes except for the change of CV at 9 and 12 months in the CG. There are, to our knowledge, no previous RCTs comparing changes in glycemic variability with the use of an app. The difference between the former observational study and the current report could be due to the well-known intrinsic bias of non-randomized studies and the lower HbA_1c_ at baseline in the present study. The direction for the additional five glucose-related outcomes was less favorable for the IG except for HbA_1c_. Overall, no major differences were observed between groups.

Adherence to follow-up visits and healthcare professional advice was good in both groups. This is especially relevant in the IG because two of the five visits were teleconsultations. Of note, the dropouts in the mySugr^®^ group were due to incompatibility of the user smartphone, while no participant withdrew from the study after initiation of the intervention. Satisfaction with the mySugr^®^ app was high (17.55 out of 20), and the patients, in support of their approval, highlighted its design, ease of use, bolus calculator, and access to glucose statistics and historical data. Moreover, all but one patient still used the app 3 months after the end of the study. This information is relevant since, currently, information on persistent utilization of apps is scarce [37].

In the present study, the use of CGM was an exclusion criterion. At study initiation in March 2019, funded access to CGM in Catalonia was limited to T1DM people with unawareness of frequent hypoglycemia. Despite the benefits of CGM and today’s rapid increase in its use, an important segment of PwD are only using BGM [38]. Thus, knowledge about tools that they can use is important.

The number of BGM tests performed by patients included in this report (3.5 at baseline in IG, 4.56 in CG) is similar to the ≥ 4 tests per day recommended by the ADA and NICE guidelines [39, 40] and also in line with reports indicating that fewer than half of PwD adhere to the recommendation of ≥ 4 tests per day [41].

The restrictions imposed by the COVID-19 pandemic taught us the extreme usefulness of easy access to a digital solution to replace a proportion of face-to-face visits without any worsening of clinical outcomes and PROMs is becoming more relevant than ever. Our findings can be applied among adults with T1DM who own a smartphone and reflect the need for apps that can be versatile and useful for successful self-management of diabetes.

The main limitation of our study is its size. We had to stop recruitment in March 2020, before achieving the calculated sample size of 33 patients, because of the COVID-19 pandemic, and three patients were excluded after randomization due to previous unknown incompatibility of the app with their smartphones.Nevertheless, this in no way diminishes the exploratory nature of the study. Second, we used simple randomization, although block randomization is more effective in ensuring balanced group sizes. Despite this, as detailed in the results section and the CONSORT diagram illustrating participant flow, we successfully enrolled 28 patients, with 14 in each group. Another limitation is that instead of using specific questionnaires, treatment satisfaction was measured with a questionnaire developed ad hoc for the present study, which was not validated for the Spanish population. The main strength of the study is the careful analysis of glucose-related outcomes and user experience.

In conclusion, patients using mySugr^®^ together with teleconsultation achieved similar results to those following usual care in empowerment, other PROMs, and most glucose-related outcomes, thus supporting the app’s use in combination with face-to-face visits.

## Electronic supplementary material

Below is the link to the electronic supplementary material.

Supplementary Material 1
