# Supplementary material for: Can a mobile application improve glucose-related and patient-reported outcome measures (PROMs) in people with type 1 diabetes mellitus? A randomized controlled trial using the mySugr® app
Source: Hormones (Athens). 2024 Oct 16;24(1):137–47. doi: 10.1007/s42000-024-00609-z (PMC11911260; doi:10.1007/s42000-024-00609-z)
Supplement: Supplementary file 1 — Supplementary Material 1 [file 42000_2024_609_MOESM1_ESM.docx]

**SUPPLEMENTAL MATERIAL**

**Can a mobile application improve glucose-related and patient-reported outcome measures (PROMS) in people with type 1 diabetes mellitus? A randomized CONTROLLED trial using mySugr® APP**

**Gemma Cuixart, M. D.^a,b*^, Rosa Corcoy, Ph. D.^a,c,d^, Cintia González, Ph. D.^c,d^**

**Author Affiliations:**

^a^Medicine Department of Universitat Autònoma de Barcelona (UAB)

Edifici M, Av. De Can Domènech

08193 Bellaterra (Spain)

935811902

^b^Research Institute of Hospital de la Santa Creu i Sant Pau

Carrer de Sant Quintí 77-79

08041 Barcelona (Spain)

935565617

^c^Endocrinology and Nutrition Department of Hospital de la Santa Creu i Sant Pau

Carrer de Sant Quintí 89

08041 Barcelona (Spain)

935565661

^d^CIBER-BBN

Avenida Monforte de Lemos, 3-5. Pabellón 11. Planta 0.

28029 Madrid (Spain)

***Corresponding author:** Gemma Cuixart

[gcuixart@santpau.cat](mailto:gcuixart@santpau.cat)

ORCID ID: 0000-0001-9217-7441

**Supplementary table 1.** CONSORT checklist for randomized trial

|  |  | Reporting Item | Page Number |
| --- | --- | --- | --- |
| **Title and Abstract** |  |  |  |
| Title | [#1a](https://www.goodreports.org/reporting-checklists/consort/info/#1a) | Identification as a randomized trial in the title. | 1 |
| Abstract | [#1b](https://www.goodreports.org/reporting-checklists/consort/info/#1b) | Structured summary of trial design, methods, results, and conclusions | 2 |
| **Introduction** |  |  |  |
| Background and objectives | [#2a](https://www.goodreports.org/reporting-checklists/consort/info/#2a) | Scientific background and explanation of rationale | 4-6 |
| Background and objectives | [#2b](https://www.goodreports.org/reporting-checklists/consort/info/#2b) | Specific objectives or hypothesis | 6 |
| **Methods** |  |  |  |
| Trial design | [#3a](https://www.goodreports.org/reporting-checklists/consort/info/#3a) | Description of trial design (such as parallel, factorial) including allocation ratio. | 7 |
| Trial design | [#3b](https://www.goodreports.org/reporting-checklists/consort/info/#3b) | Important changes to methods after trial commencement (such as eligibility criteria), with reasons | no |
| Participants | [#4a](https://www.goodreports.org/reporting-checklists/consort/info/#4a) | Eligibility criteria for participants | 7 |
| Participants | [#4b](https://www.goodreports.org/reporting-checklists/consort/info/#4b) | Settings and locations where the data were collected | 7 |
| Interventions | [#5](https://www.goodreports.org/reporting-checklists/consort/info/#5) | The experimental and control interventions for each group with sufficient details to allow replication, including how and when they were actually administered | 7-8 |
| Outcomes | [#6a](https://www.goodreports.org/reporting-checklists/consort/info/#6a) | Completely defined prespecified primary and secondary outcome measures, including how and when they were assessed | 8-11 |
| Sample size | [#7a](https://www.goodreports.org/reporting-checklists/consort/info/#7a) | How sample size was determined. | 11 |
| Sample size | [#7b](https://www.goodreports.org/reporting-checklists/consort/info/#7b) | When applicable, explanation of any interim analyses and stopping guidelines | n/a |
| Randomization - Sequence generation | [#8a](https://www.goodreports.org/reporting-checklists/consort/info/#8a) | Method used to generate the random allocation sequence. | 8 |
| Randomization - Sequence generation | [#8b](https://www.goodreports.org/reporting-checklists/consort/info/#8b) | Type of randomization; details of any restriction (such as blocking and block size) | 8 |
| Randomization - Allocation concealment mechanism | [#9](https://www.goodreports.org/reporting-checklists/consort/info/#9) | Mechanism used to implement the random allocation sequence (such as sequentially numbered containers), describing any steps taken to conceal the sequence until interventions were assigned | 8 |
| Randomization - Implementation | [#10](https://www.goodreports.org/reporting-checklists/consort/info/#10) | Who generated the allocation sequence, who enrolled participants, and who assigned participants to interventions | 8 |
| Blinding | [#11a](https://www.goodreports.org/reporting-checklists/consort/info/#11a) | If done, who was blinded after assignment to interventions (for example, participants, care providers, those assessing outcomes) and how. | n/a |
| Blinding | [#11b](https://www.goodreports.org/reporting-checklists/consort/info/#11b) | If relevant, description of the similarity of interventions | n/a |
| Statistical methods | [#12a](https://www.goodreports.org/reporting-checklists/consort/info/#12a) | Statistical methods used to compare groups for primary and secondary outcomes | 11 |
| Statistical methods | [#12b](https://www.goodreports.org/reporting-checklists/consort/info/#12b) | Methods for additional analyses, such as subgroup analyses and adjusted analyses | 11 |
| Outcomes | [#6b](https://www.goodreports.org/reporting-checklists/consort/info/#6b) | Any changes to trial outcomes after the trial commenced, with reasons | no |
| **Results** |  |  |  |
| Participant flow diagram (strongly recommended) | [#13a](https://www.goodreports.org/reporting-checklists/consort/info/#13a) | For each group, the numbers of participants who were randomly assigned, received intended treatment, and were analysed for the primary outcome | 12 |
| Participant flow | [#13b](https://www.goodreports.org/reporting-checklists/consort/info/#13b) | For each group, losses and exclusions after randomization, together with reason | 12 |
| Recruitment | [#14a](https://www.goodreports.org/reporting-checklists/consort/info/#14a) | Dates defining the periods of recruitment and follow-up | 10 |
| Recruitment | [#14b](https://www.goodreports.org/reporting-checklists/consort/info/#14b) | Why the trial ended or was stopped | 12 |
| Baseline data | [#15](https://www.goodreports.org/reporting-checklists/consort/info/#15) | A table showing baseline demographic and clinical characteristics for each group | 26 |
| Numbers analysed | [#16](https://www.goodreports.org/reporting-checklists/consort/info/#16) | For each group, number of participants (denominator) included in each analysis and whether the analysis was by original assigned groups | 27 |
| Outcomes and estimation | [#17a](https://www.goodreports.org/reporting-checklists/consort/info/#17a) | For each primary and secondary outcome, results for each group, and the estimated effect size and its precision (such as 95% confidence interval) | 27 |
| Outcomes and estimation | [#17b](https://www.goodreports.org/reporting-checklists/consort/info/#17b) | For binary outcomes, presentation of both absolute and relative effect sizes is recommended | n/a |
| Ancillary analyses | [#18](https://www.goodreports.org/reporting-checklists/consort/info/#18) | Results of any other analyses performed, including subgroup analyses and adjusted analyses, distinguishing pre-specified from exploratory | n/a |
| Harms | [#19](https://www.goodreports.org/reporting-checklists/consort/info/#19) | All important harms or unintended effects in each group (For specific guidance see CONSORT for harms) | 13 |
| **Discussion** |  |  |  |
| Limitations | [#20](https://www.goodreports.org/reporting-checklists/consort/info/#20) | Trial limitations, addressing sources of potential bias, imprecision, and, if relevant, multiplicity of analyses | 17 |
| Interpretation | [#22](https://www.goodreports.org/reporting-checklists/consort/info/#22) | Interpretation consistent with results, balancing benefits and harms, and considering other relevant evidence | 14-17 |
| Registration | [#23](https://www.goodreports.org/reporting-checklists/consort/info/#23) | Registration number and name of trial registry | 2, 7 |
| Generalisability | [#21](https://www.goodreports.org/reporting-checklists/consort/info/#21) | Generalisability (external validity, applicability) of the trial findings | 16-17 |
| **Other information** |  |  |  |
| Interpretation | [#22](https://www.goodreports.org/reporting-checklists/consort/info/#22) | Interpretation consistent with results, balancing benefits and harms, and considering other relevant evidence | 14-17 |
| Registration | [#23](https://www.goodreports.org/reporting-checklists/consort/info/#23) | Registration number and name of trial registry | 2, 7 |
| Protocol | [#24](https://www.goodreports.org/reporting-checklists/consort/info/#24) | Where the full trial protocol can be accessed, if available | 2, 7 |
| Funding | [#25](https://www.goodreports.org/reporting-checklists/consort/info/#25) | Sources of funding and other support (such as supply of drugs), role of funders | 18 |

**Supplementary table 2.** Diabetes Empowerment Scale-Short Form questionnaire Spanish version (DES-SF-S)

| En general creo que yo: | Muy de  acuerdo | De  acuerdo | Ni de  acuerdo  ni en  desacuerdo | En desacuerdo | Muy en  desacuerdo |
| --- | --- | --- | --- | --- | --- |
| **1. Conozco con qué partes del cuidado de mi diabetes estoy**  **insatisfecha(o)** |  |  |  |  |  |
| **2. Soy capaz de convertir mis metas en un plan de acción práctico y concreto** |  |  |  |  |  |
| **3. Puedo intentar hacer diferentes cosas para superar las barreras enfrento para lograr mis metas** |  |  |  |  |  |
| **4. Puedo decir cómo me estoy sintiendo viviendo con la diabetes** |  |  |  |  |  |
| **5. Conozco maneras positivas que uso para enfrentar el estrés que me causa la diabetes** |  |  |  |  |  |
| **6. Conozco en dónde puedo encontrar apoyo para vivir y cuidar de mi diabetes** |  |  |  |  |  |
| **7. Conozco lo que me ayuda a permanecer motivado para cuidar de mi diabetes** |  |  |  |  |  |
| **8. Me conozco lo suficiente como persona como para tomar las**  **decisiones que me convienen para el cuidado de mi diabetes** |  |  |  |  |  |

**Supplementary table 3.** Diabetes-related tasks questionnaire

1. **¿Considera que el tiempo que invierte en la gestión de su diabetes es adecuado?**

1= Nada adecuado 2= Poco adecuado 3= Adecuado 4= Muy adecuado

1. **¿Cuánto tiempo medio invierte al día en el control de su diabetes?**

1= >30 min 2= 20-30 min 3= 10-20 min 4= <10 min

1. **¿Cuántas acciones de media realiza al día en relación a su diabetes?**

1= >15 2= 10-15 3= 5-10 4= <5

1. **¿Las herramientas de las que dispone actualmente le ayudan en la gestión de su diabetes?**

1= Nada 2= Poco 3= Suficiente 4= Mucho

1. **¿Las herramientas de las que dispone actualmente le ayudan a reducir el número de tareas relacionadas con su diabetes?**

1= Nada 2= Poco 3= Suficiente 4= Mucho

**Supplementary table 4.** Diabetes Quality of Life questionnaire Spanish version (EsDQOL)

**Satisfacción:** 1 = muy satisfecho, 2 = bastante satisfecho, 3 = algo satisfecho, 4 = poco satisfecho y 5 = nada satisfecho

1. ¿Está usted satisfecho con la cantidad de tiempo que tarda en controlar su diabetes?

2. ¿Está usted satisfecho con la cantidad de tiempo que ocupa en revisiones?

3. ¿Está usted satisfecho con el tiempo que tarda en determinar su nivel de azúcar?

4. ¿Está usted satisfecho con su tratamiento actual?

5. ¿Está usted satisfecho con la flexibilidad que tiene en su dieta?

6. ¿Está usted satisfecho con la carga que supone su diabetes en su familia?

7. ¿Está usted satisfecho con su conocimiento sobre la diabetes?

8. ¿Está usted satisfecho con su sueño?

9. ¿Está usted satisfecho con sus relaciones sociales y amistades?

10. ¿Está usted satisfecho con su vida sexual?

11. ¿Está usted satisfecho con sus actividades en el trabajo, colegio u hogar?

12. ¿Está usted satisfecho con la apariencia de su cuerpo?

13. ¿Está usted satisfecho con el tiempo que emplea haciendo ejercicio?

14. ¿Está usted satisfecho con su tiempo libre?

15. ¿Está usted satisfecho con su vida en general?

**Impacto:** 1 = nunca, 2 = casi nunca, 3 = a veces, 4 = casi siempre y 5 = siempre

16. ¿Con qué frecuencia siente dolor asociado con el tratamiento de su diabetes?

17. ¿Con qué frecuencia se siente avergonzado por tener que tratar su diabetes en público?

18. ¿Con qué frecuencia se siente físicamente enfermo?

19. ¿Con qué frecuencia su diabetes interfiere en su vida familiar?

20. ¿Con qué frecuencia tiene problemas para dormir?

21. ¿Con qué frecuencia encuentra que su diabetes limita sus relaciones sociales y amistades?

22. ¿Con qué frecuencia se siente restringido por su dieta?

23. ¿Con qué frecuencia su diabetes interfiere en su vida sexual?

24. ¿Con qué frecuencia su diabetes le impide conducir o usar una máquina (p. ej. máquina de escribir)?

25. ¿Con qué frecuencia su diabetes interfiere en la realización de ejercicio?

26. ¿Con qué frecuencia abandona sus tareas en el trabajo, colegio o casa por su diabetes?

27. ¿Con qué frecuencia se encuentra usted mismo explicándose qué significa tener diabetes?

28. ¿Con qué frecuencia cree que su diabetes interrumpe sus actividades de tiempo libre?

29. ¿Con qué frecuencia bromean con usted por causa de su diabetes?

30. ¿Con qué frecuencia siente que por su diabetes va al cuarto de baño más que los demás?

31. ¿Con qué frecuencia come algo que no debe antes de decirle a alguien que tiene diabetes?

32. ¿Con qué frecuencia esconde a los demás el hecho de que usted está teniendo una reacción insulínica?

**Preocupación social/vocacional:** 1 = nunca, 2 = casi nunca, 3 = a veces, 4 = casi siempre y 5 = siempre

33. ¿Con qué frecuencia le preocupa si se casará?

34. ¿Con qué frecuencia le preocupa si tendrá hijos?

35. ¿Con qué frecuencia le preocupa si conseguirá el trabajo que desea?

36. ¿Con qué frecuencia le preocupa si le será denegado un seguro?

37. ¿Con qué frecuencia le preocupa si será capaz de completar su educación?

38. ¿Con qué frecuencia le preocupa si perderá el empleo?

39. ¿Con qué frecuencia le preocupa si podrá ir de vacaciones o de viaje?

**Preocupación relacionada con la diabetes:** 1 = nunca, 2 = casi nunca, 3 = a veces, 4 = casi siempre y 5 = siempre

40. ¿Con qué frecuencia le preocupa si perderá el conocimiento?

41. ¿Con qué frecuencia le preocupa que su cuerpo parezca diferente a causa de su diabetes?

42. ¿Con qué frecuencia le preocupa si tendrá complicaciones debidas a su diabetes?

43. ¿Con qué frecuencia le preocupa si alguien no saldrá con usted a causa de su diabetes?

**Supplementary table 5**. Diabetes Distress Scale questionnaire Spanish version (DSS-S)

INSTRUCCIONES: Vivir con diabetes a veces es difícil. Habrá numerosos problemas referentes a la diabetes que puedan variar en severidad. Estos problemas pueden variar de grado, algunos pueden ser más graves que otros. Enumerados abajo, hay 17 posibles problemas que las personas con diabetes puedan enfrentar. Considere hasta qué grado le han afligido cada una de las siguientes situaciones DURANTE EL ULTIMO MES, y por favor haga un círculo alrededor del número apropiado.

Por favor, manténgase al tanto que le estamos pidiendo que indique el grado de severidad en el cual, uno de estos elementos le complica la vida, NO simplemente si se aplica a usted. Si determina que algún elemento en particular, no es una molestia ni problema para usted, circularía el “1”. Si es severamente molesto, circularía el “6”.

|  | No es un  problema | Es un  pequeño  problema | Es un  problema  moderado | Es un  problema  algo  grave | Es un  problema  grave | Es un  problema  muy  grave |
| --- | --- | --- | --- | --- | --- | --- |
| **1. Sentirme agotado mental y físicamente por el esfuerzo constante para controlar la diabetes** | 1 | 2 | 3 | 4 | 5 | 6 |
| **2. Sentir que mi doctor no sabe lo suficiente acerca de la diabetes y del cuidado de la diabetes** | 1 | 2 | 3 | 4 | 5 | 6 |
| **3. No sentir confianza en mi habilidad para manejar mi diabetes día a día** | 1 | 2 | 3 | 4 | 5 | 6 |
| **4. Sentirme enojado(a), asustado(a), o deprimido(a) cuando pienso en el vivir con diabetes** | 1 | 2 | 3 | 4 | 5 | 6 |
| **5. Sentir que mi doctor no me da recomendaciones lo suficientemente específicas para controlar mi diabetes** | 1 | 2 | 3 | 4 | 5 | 6 |
| **6. Sentir que no me estoy analizando la sangre con suficiente frecuencia** | 1 | 2 | 3 | 4 | 5 | 6 |
| **7. Sentir que haga lo que haga, siempre tendré complicaciones serias a largo plazo** | 1 | 2 | 3 | 4 | 5 | 6 |
| **8. Sentir que fracaso a menudo con mi régimen de diabetes** | 1 | 2 | 3 | 4 | 5 | 6 |
| **9. Sentir que ni mis amigos ni mi familia me dan suficiente apoyo en mis esfuerzos para**  **cuidarme (planean actividades que chocan con mi horario, me animan a comer comidas**  **"impropias"** | 1 | 2 | 3 | 4 | 5 | 6 |
| **10. Sentir que la diabetes**  **controla mi vida** | 1 | 2 | 3 | 4 | 5 | 6 |
| **11. Sentir que mi doctor no toma en serio mis**  **preocupaciones** | 1 | 2 | 3 | 4 | 5 | 6 |
| **12. Sentir que no estoy**  **manteniendo un régimen**  **dietético saludable** | 1 | 2 | 3 | 4 | 5 | 6 |
| **13. Sentir que ni mis amigos ni mi familia saben lo difícil que es vivir con la diabetes** | 1 | 2 | 3 | 4 | 5 | 6 |
| **14. Sentirse abrumado(a) por la atención que requiere vivir con la diabetes** | 1 | 2 | 3 | 4 | 5 | 6 |
| **15. Sentir que no tengo un doctor a quién puedo ver con la frecuencia suficiente para discutir mi diabetes** | 1 | 2 | 3 | 4 | 5 | 6 |
| **16. Sentir que no tengo la**  **motivación necesaria para**  **controlar mi diabetes** | 1 | 2 | 3 | 4 | 5 | 6 |
| **17. Sentir que ni mis amigos ni mi familia me dan el apoyo emocional que me gustaría tener** | 1 | 2 | 3 | 4 | 5 | 6 |

**Supplementary table 6.** mySugr® app satisfaction questionnaire

Puntúe, por favor, de 0 a 5 el nivel de satisfacción en cada pregunta. Siendo 0 la puntuación más baja y por tanto la de menor satisfacción y 5 la más alta y la de mayor satisfacción

1. **¿Considera que el manejo de la aplicación es fácil e intuitiva?**

| 0 | 1 | 2 | 3 | 4 | 5 |
| --- | --- | --- | --- | --- | --- |

1. **¿Le ha ayudado la App en el manejo de su diabetes?**

| 0 | 1 | 2 | 3 | 4 | 5 |
| --- | --- | --- | --- | --- | --- |

1. **¿El entrenamiento que ha recibido en su utilización sido ha sido suficiente?**

| 0 | 1 | 2 | 3 | 4 | 5 |
| --- | --- | --- | --- | --- | --- |

1. **¿El tiempo empleado al día en el uso de la App lo considera adecuado?**

| 0 | 1 | 2 | 3 | 4 | 5 |
| --- | --- | --- | --- | --- | --- |

1. **¿Qué eliminaría de la App?**
2. **¿Qué añadiría a la App**
